# Co-Localization of Crotamine with Internal Membranes and Accentuated Accumulation in Tumor Cells

**DOI:** 10.3390/molecules23040968

**Published:** 2018-04-20

**Authors:** Nicole Caroline Mambelli-Lisboa, Juliana Mozer Sciani, Alvaro Rossan Brandão Prieto da Silva, Irina Kerkis

**Affiliations:** 1 Laboratory of Genetics, Butantan Institute, Sao Paulo 05503-900, Brazil; alvaro.prieto@butantan.gov.br; 2 CENTD—Center of Excellence in New Target Discovery, Butantan Institute, Sao Paulo 05503-900, Brazil; juliana.sciani@butantan.gov.br; 3 Biochemistry and Biophysics Laboratory, Butantan Institute, Sao Paulo 05503-900, Brazil

**Keywords:** co-localization, molecular imaging, membrane trafficking, cell penetrating peptide (CPP), crotamine, tumor marker

## Abstract

Crotamine is a highly cationic; cysteine rich, cross-linked, low molecular mass cell penetrating peptide (CPP) from the venom of the South American rattlesnake. Potential application of crotamine in biomedicine may require its large-scale purification. To overcome difficulties related with the purification of natural crotamine (nCrot) we aimed in the present study to synthesize and characterize a crotamine analog (sCrot) as well investigate its CPP activity. Mass spectrometry analysis demonstrates that sCrot and nCrot have equal molecular mass and biological function—the capacity to induce spastic paralysis in the hind limbs in mice. sCrot CPP activity was evaluated in a wide range of tumor and non-tumor cell tests performed at different time points. We demonstrate that sCrot-Cy3 showed distinct co-localization patterns with intracellular membranes inside the tumor and non-tumor cells. Time-lapse microscopy and quantification of sCrot-Cy3 fluorescence signalss in living tumor versus non-tumor cells revealed a significant statistical difference in the fluorescence intensity observed in tumor cells. These data suggest a possible use of sCrot as a molecular probe for tumor cells, as well as, for the selective delivery of anticancer molecules into these tumors.

## 1. Introduction

Crotamine, composed of 42 amino acids, is a myo-neurotoxin derived from *Crotalus durissus terrificus* venom that belongs to the reptilian β-defensins—a group of small cationic antimicrobial peptides—that present high sequence variability preservation and the same three-dimensional structure. Crotamine was identified as a cell penetrating peptide (CPP) which demonstrates specificity for actively proliferating cells, interacting with different intracellular targets [1,2,3,4]. Cationic CPPs are short arginine and lysine rich positively charged sequences [5,6]. They can penetrate usually impermeable cell membranes and may trigger actions in the cytoplasm or the nucleus of cells, or both [7,8,9,10,11,12,13]. 

Successful achievements over the past years with the use of CPPs in various preclinical models have revealed their remarkable potential for clinical application [14]. Despite the great potential of CPPs as a new therapeutic strategy, a limitation is emergent, due to the lack of selectivity of CPPs for specific cell types or cell organelles. This is a major obstacle to the clinical application of CPPs as, for instance, a method for cancer targeting for diagnostic probe imaging or even for the delivery of therapeutic drugs into tumor sites [6]. In this regard, toxin-derived CPPs seem to be an exception that proves the rule [15,16,17,18,19,20,21,22]. Supplemental Table S1 lists natural CPP toxins and their mechanism of action in vitro and in vivo at the cell level, as well as their possible intracellular targets [1,15,16,17,18,19,21,23,24,25,26,27,28,29,30,31]. 

The development and investigation of novel therapeutic molecules obtained from natural sources seem to be a challenging scientific problem for pharmacology. Despite the promising therapeutic effects of natural peptides and proteins derived from snake venoms, their purification and preparation in large amounts is difficult, especially when the involve three disulfide bonds. Moreover, synthetic analogs of natural peptides, generally, contain only natural amino acids in their composition, have no natural variability and have fewer side effects. Therefore, synthetic peptides have considerable advantage over natural molecules, especially in clinical studies [32], as well as, because of the welfare of wild and captivity rattlesnakes [33,34]. 

To date, little is known about the interaction of crotamine with intracellular membranes. Our study is the first step to discover sCrot (synthetic crotamine) potential intracellular molecular targets aiming at establishing its biotechnological applications. Such a protein was correctly synthesized and structured, maintaining native crotamine’s YKQCHKKGGHCFPKEKICLPPSSDFGKMDCRWRWK CCKKGSG amino acid sequence, as wells its three-disulfide bonds (Cys4-Cys36, Cys11-Cys30, Cys18-Cys37). 

We verified sCrot’s molecular mass and its capacity to induce spastic paralysis in the hind limbs in mice as observed in its natural homolog (nCrot). Next, sCrot uptake in a wide range of tumor cells was evaluated at different time points, in comparison with non-tumor cells. We also investigated sCrot co-localization with internal membranes in tumor versus non-tumor fixed cells. Time-lapse fluorescence microscopy was used to examine sCrot penetration into living tumor versus non-tumor cells and to quantify its efficiency in both cell types, by measuring the fluorescence signal intensity. Additionally, the effect of different sCrot concentrations on tumor and non-tumor cell viability has been evaluated. 

## 2. Results

### 2.1. Comparison of sCrot and nCrot

This investigation demonstrates that both have equal molecular mass, in vivo biological response and similar CPP activities (Supplementary Figure S1). The CPP activity of sCrot reported here was extensively investigated in different cell types, whether tumor or not, at different time points, concentrations and in two and three dimension models.

### 2.2. sCrot-Cy3 Uptake

**Figure 1:**
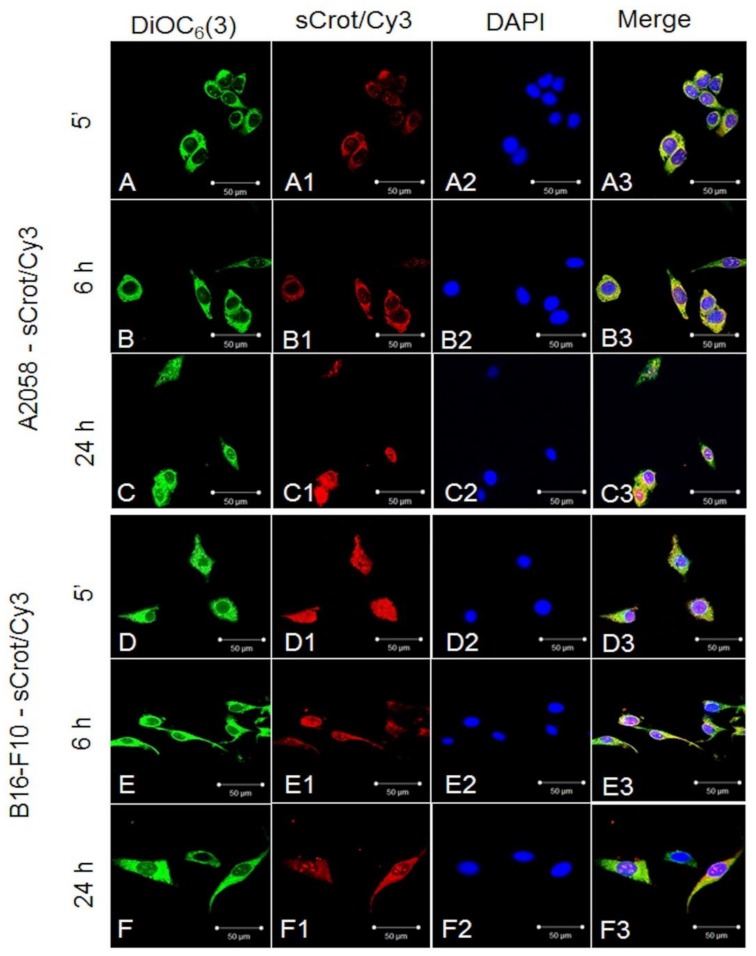
sCrot-Cy3 uptake and co-localization with internal cellular membranes stained by DiOC_6_(3) observed in tumor cells. (**A**–**A3**) sCros-Cy3 uptake after 5 min in human A2058 cells (**A**) Internal cellular membranes stained by DiOC_6_(3) (green); (**A1**) sCrot-Cy3 uptake; (**A3**) Nuclei stained by DAPI; (**A3**) Merged image of **A** + **A1** + **A2**. (**B**–**B3**, **C**–**C3**) same as in (**A**–**A3**) showing sCrot-Cy3 uptake after 6 and 24 h respectively. (**D**–**D3**, **E**–**E3**, **F**–**F3**) same as in (**A**–**A3**, **B**–**B3**, **C**–**C3**) demonstrating sCrot-Cy3 uptake after 5 min, 6 and 24 h respectively in murine B16-F10 cells. Scale bars (**A**–**F3**) = 50 µm. (**A**–**F3**) = Fluorescent confocal microscopy (FCM).

**Figure 2:**
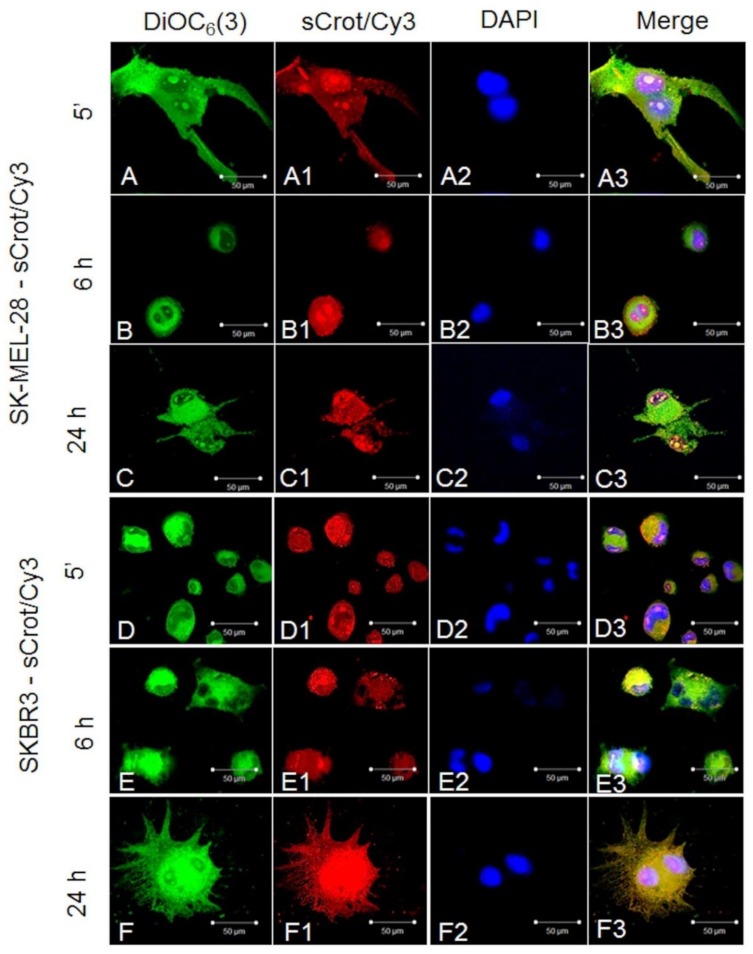
sCrot-Cy3 uptake and co-localization with internal cellular membranes stained by DiOC_6_(3) observed in tumor cells. (**A**–**A3**) sCrot-Cy3 uptake after 5 min in human SK-MEL-28 cells (**A**) Internal cellular membranes stained by DiOC_6_(3) (green); (**A1**) sCrot-Cy3 uptake; (**A3**) Nuclei stained by DAPI; (**A3**) Merged image of **A** + **A1** + **A2**. (**B**–**B3**, **C**–**C3**) same as in (**A**–**A3**) showing sCrot-Cy3 uptake after 6 and 24 h respectively. (**D**–**D3**, **E**–**E3**, **F**–**F3**) same as in (**A**–**A3**, **B**–**B3**, **C**–**C3**) demonstrating sCrot-Cy3 uptake after 5 min, 6 and 24 h respectively in human SKBR3 cells. Scale bars (**A**–**F3**) = 50 µm. (**A**–**F3**) = Fluorescent confocal microscopy (FCM).

**Figure 3:**
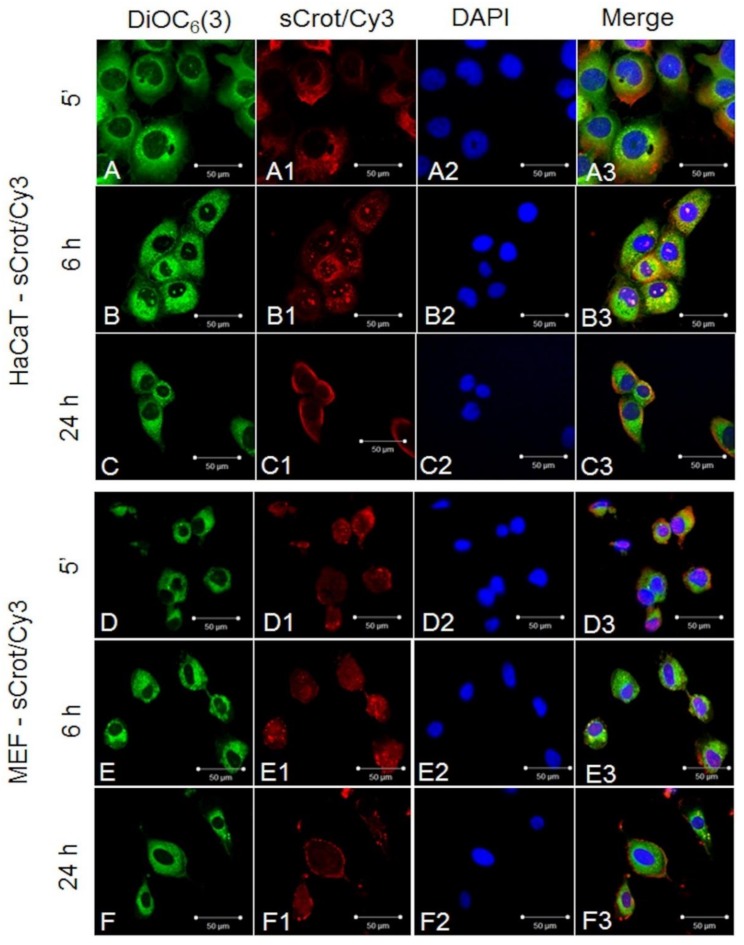
sCrot-Cy3 uptake and co-localization with internal cellular membranes stained by DiOC_6_(3) observed in non-tumor cells. (**A**–**A3**) sCrot-Cy3 uptake after 5 min in human HaCaT cells (**A**) Internal cellular mebranes stained by DiOC_6_(3) (green); (**A1**) sCrot-Cy3 uptake; (**A3**) Nuclei stained by DAPI; (**A3**) Merged image of **A** + **A1** + **A2**. (**B**–**B3**, **C**–**C3**) same as in (**A**–**A3**) showing sCrot-Cy3 uptake after 6 and 24 h respectively. (**D**–**D3**, **E**–**E3**, **F**–**F3**) same as in (**A**–**A3**, **B**–**B3**, **C**–**C3**) demonstrating sCros uptake after 5 min, 6 and 24 h respectively in murine MEF cells. Scale bars (**A**–**F3**) = 50 µm. (**A**–**F3**) = Fluorescent confocal microscopy (FCM).

**Figure 4:**
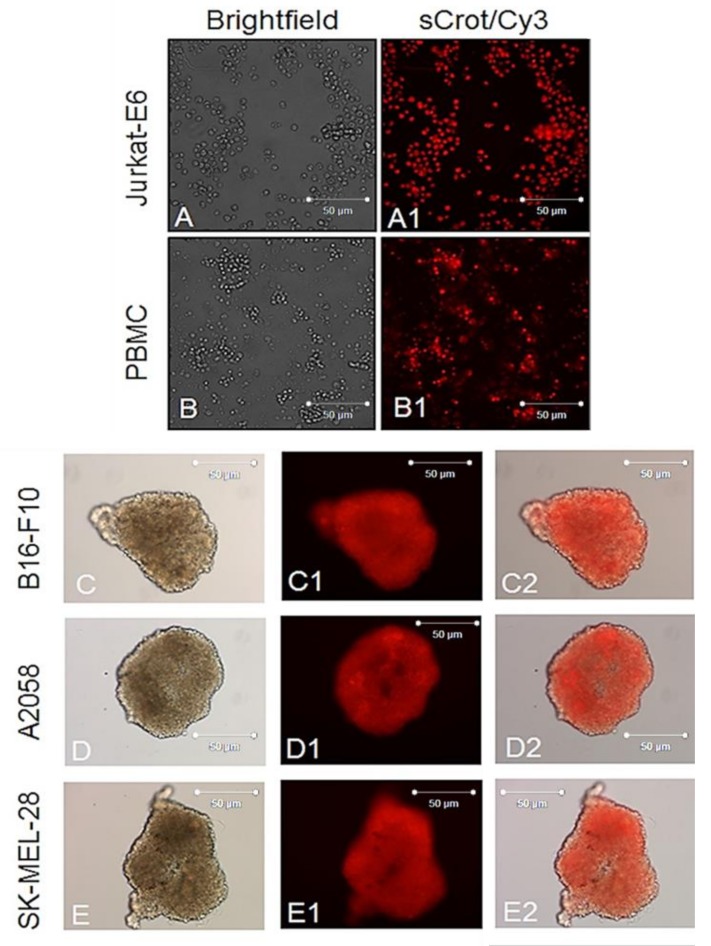
sCrot-Cy3 uptake observed in cells growing in suspension and in melanomaspheres. (**A**–**A2**) sCrot uptake in Jurkat-E6 cells after 5 min; (**A**) Phase contrast (PC); (**A1**) sCrot-Cy3; (**B**, **B1**) same as in (**A**–**A2**) observed in PBMC. (**C**–**C2**) sCrot-Cy3 uptake after 5 min in melanospheres derived from B16-F10 cells. (**D**–**D2**, **E**–**E2**) same as in (**C**–**C2**) showed in melanospheres derived from A2058 and SK-MEL-28, respectively. (**A**–**E**) = PC. (**A**–**B2**, **C1**–**E1**) = Epi-fluorescence. (**C2**–**E2**) Merged images between (**C**, **C1**), (**D**, **D1**) and (**E**, **E1**). Magnification (**A**–**B2**) = 40x (**C**–**E2**) = 20x. Scale bars (**A**–**F3**) = 50 µm.

First, sCrot-Cy3 uptake was investigated in human melanoma cells A2058 and SK-MEL-85, murine melanoma (B16-F10), mammary tumor (SKBR3), human T lymphocytes from leukemia (Jurkat-E6), mononuclear human cells (PBMC), embryonic murine fibroblasts (MEF) and human keratinocytes (HaCaT). The sCrot-Cy3 demonstrated the ability to penetrate rapidly into all studied cells, showing, however, tumor cells strong preference. The uptake dynamics demonstrated that sCrot penetrates within 5 min and it is still present in the cells after 6 and 24 h of incubation, showing preferential intracellular localization for each cell line over time (Figure 1, Figure 2, Figure 3 and Figure 4A,B). To verify the interaction of sCrot-Cy3 with internal cell membranes, the fluorescent dye DiOC_6_(3) was used. The co-localization between sCrot-Cy3 with internal cell membranes was observed by the fusion images as shown on Figure 1, Figure 2 and Figure 3 in the panels A3–F3. Such interaction was not investigated for PBMC and Jurkat-E6 cells, since that these cells morphology or their small sizes hinder the confocal analyses.

### 2.3. Uptake of sCrot-Cy3 in 3-D Models 

Next sCrot-Cy3 CPP capacity to penetrate in the cells organized in a three-dimensional (3-D) model that mimics an in vivo environment was investigated [35]. Melanoma cells are known to form spheroids (melanospheres). In turn, melanospheres are enriched in cells with high clonogenic potential, reflecting self-renewal capacity for of tumor stem cells [36]. After 15 days sCrot-Cy3 at final concentration 1 μM was added onto culture medium with melanospheres derived from all studied melanoma cell lines and its internalization were analyzed by fluorescence microscopy after 5 min. Figure 4C–E shows rapid uptake, robust and homogeneous distribution of sCrot-Cy3 in the cells within melanospheres.

### 2.4. Live-Cell Imaging by Time-Lapse Microscopy 

**Figure 5:**
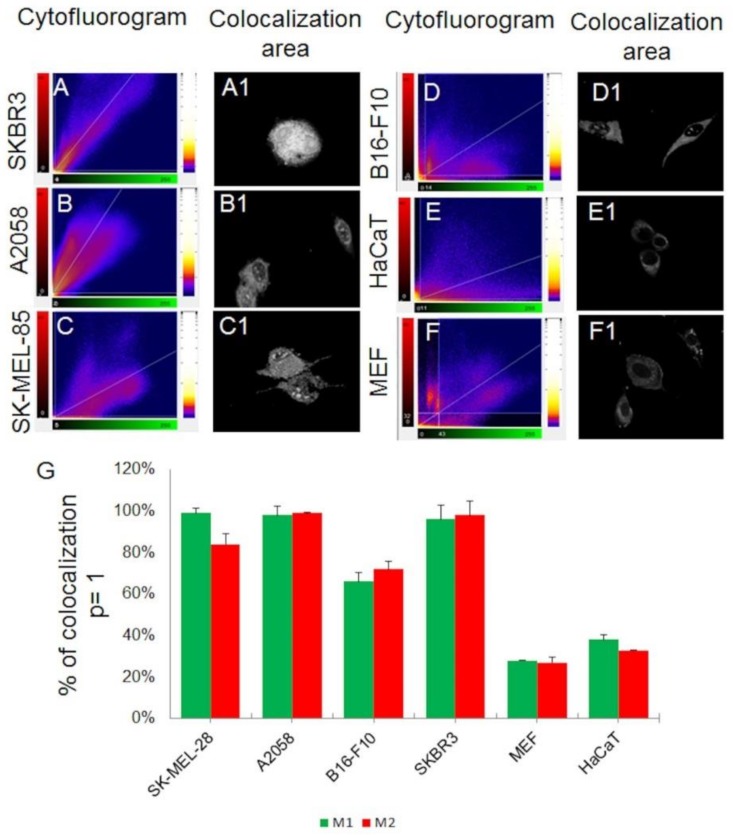
Cytofluorograms show distribution of pixels and intensity correlation quotient—ICQ in images according to selected pair of channels (sCrot-Cy3: red; DiOC_6_(3): green). (**A**, **A1**) Colocalized pinkish pixels are located along to the diagonal channel showed by SKBR3 cells; same as in (**B**, **B1**) and (**C**, **C1**) showed by A2058 and SK-MEL-28 cells respectively; (**D**, **D1**) Semi-Colocalized pinkish pixels are split, “two tailed” shape of scatter gram; (**E**, **E1**) and (**F**, **F1**) shows the lowest percentage of colocalized pixels presenting different intensities for both channels. (**G**) shows the percentage of co-localization measured by Mander’coef with Costes (≥95%) and Pearson’s correlation coefficients (PCCs) = 1. Error bars indicate standard error from triplicate analysis.

The observation of co-localization between internal cell membranes and sCrot-Cy3 was quantified by the BioImage XD software, using the methods of Pearson [37], Manders [38], Costes [39] and Li [40]. The Pearson coefficient is an accurate measure of co-localization when the densities of the two species of interest are approximately equal [41,42]. For the two data sets of the image of interest, the intensities in the detection channel are calculated, summing all the pixels. The probability of significance is represented by the p-value using Costes. The analysis of the coefficients of Manders, M1 and M2, is performed with automatic determination of the co-localization limit values, as a function of the total density, the density between the two detection channels and the interaction fraction, such analysis is represented in percentage result between the co-localization of the two channels of interest. Finally, the Li method indicated intensity correlation—ICQ (Intensity Correlation Quotient). Supplemental Table S2 exemplifies all methods used for this analysis. The M1 coefficient is represented by DiOC_6_(3), indicating internal cell membranes and M2 by Cy3 fluorescence, indicating sCrot. As a result (Figure 5G), a high percentage (>80%) of co-localization between sCrot-Cy3 and internal cell membranes in all human tumor cells is noted, in contrast, in non-tumor cells the result of such co-localization is <40%. The areas of co-localization between the two components are measured and the ICQ is generated and presented in cytofluorograms (Figure 5A–F). It is important to note that such co-localization were performed from 3D images of confocal microscopy and several focal slices (≥4) were explored, resulting in a high significance of the analysis. The cytofluorograms (Figure 5A–F) also indicate a low fluorescence noise ratio, allowing an ideal correlation analysis between the two measured fluorescence channels [43]. 

### 2.5. sCrot-Cy3 Fluorescence Intensity and Cell Viability 

**Figure 6:**
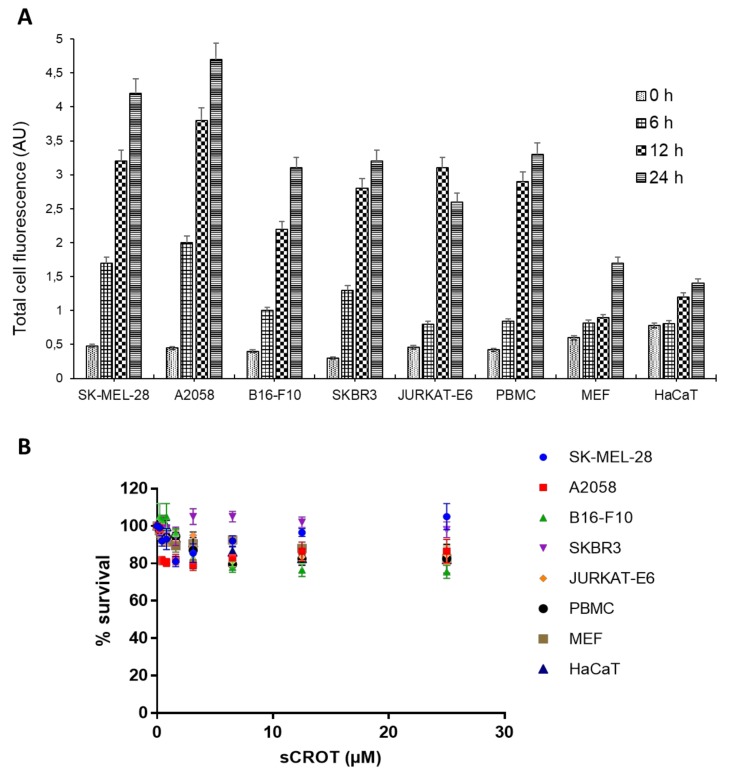
sCrot-Cy3 fluorescence intensity and cell viability after sCrot treatment. (**A**) Mean values of staining intensity in arbitrary florescence units (a.u.) and their associated standard deviations are shown for sCrot-Cy3 in the panel of tumor and non-tumor cells described. Error bars indicate standard error from triplicate analysis of different points from a total of 144 images analyzed for each cell line using ImageJ software; (**B**) Effect of sCrot on cell survival in all cell lines described for 24 h. The cell number at the control without sCrot addition was considered to be 100%. The assay was conducted three times with three replicates each time. Error bars indicate standard error from triplicates in the experiments (*n* = 3).

In the scope of the CPP activity of sCrot, its penetration into living cells in real time by time-lapse (see Supplemental Videos) was also investigated. Sequential images demonstrate constant increasing of fluorescent signal emitted by sCrot-Cy3, observed in A2058 and Sk-MEL-28 cells (Supplemental Videos S1 and S2, respectively, and Figure 6A), with its accumulation over time. Non-tumor cells—HaCaT and MEF, show a low and stable fluorescent signal during the period tested (Supplemental Videos S5 and S6, respectively, and Figure 6A). B16-F10 and SKBR3 lines, both demonstrate similar intensity of fluorescent signal emitted by sCrot-Cy3 (Supplemental Videos S3 and S4, respectively, and Figure 6A), demonstrating high intensity, however, did not show accumulation of sCrot-Cy3 in the end of the time explored. 

In addition, the CPP activity of sCrot in non-adherent cells, PBMC and Jurkat-E6 cells was also investigated using time-lapse microscopy. The Supplemental Videos S7 and S8, show quick sCrot-Cy3 internalization by these cells, which persists during the experimental period. Figure 6A shows the quantification of different patterns from the fluorescent intensity observed in this data, such quantification corroborates with the sequential images from Supplemental Videos S1–S8 of all cells studied. Following the sequences of acquired images by time-lapse microscopy, the intensity of fluorescence in respect of time of sCrot-Cy3 permanence inside PBMC and Jurkat-E6 cells (Supplemental Videos S8 and S9) was quantified, which revealed that intensity and dynamics of fluorescent signal in PBMC and Jurkat-E6 was similar to that observed in B16-F10 and SKBR3 lines (Figure 6A). 

Finally, we evaluated the viability of all studied cells after long (24 h) exposure to sCrot treatment, since it is known that nCrot has low toxicity [1]. We showed that sCrot did not influence the tested cells’ viability at the doses used in the present study and even demonstrate low cytotoxicity at higher doses (Figure 6B).

## 3. Discussion

It is known that nCrot is a non-abundant component of *Crotalus durissus terrificus* venom, and its presence in the venom depends on the geographical distribution of the snake [44]. Another problem is the separation of the nCrot from the whole venom due to the presence of other toxins that have similar molecular mass, making an efficient nCrot purification difficult. On the other hand, taking in consideration the biotechnological potential of this molecule, it is of interest to produce sCrot, which has all activity and multifunctionality of nCrot [22]. 

Herein, for the first time, we showed that sCrot shares a similar molecular mass, measured by mass spectrometry, in vivo biological function (see the Supplemental Material) and cell penetration capacity with nCrot. Relevant data was obtained regarding penetrating capacity as well as regarding intracellular membrane targeting of sCrot in tumor versus non-tumor cells. The study on sCrot potential intracellular targets revealed differential patterns of sCrot-Cy3 co-localization with markers of intracellular membranes inside the fixed tumor and non-tumoral cells. Quantification demonstrated a 2-fold higher co-localization of sCrot with intracellular membranes in tumor cells. Time-lapse microscopy and quantification of sCrot-Cy3 fluorescence signals in living tumor versus non-tumor cells, highlighted a significant statistical difference in fluorescence intensity observed in tumor cells. According to the fluorescence intensity emitted by sCrot-Cy3 over time (total 24 h) three different patterns can be distinguished: (a) high intensity, which was observed in SK-Mel28 and A2058; (b) stable intensity, which are registered in B16-F10, SKBR3, Jurkat E6 and PBMC and maintained over time and (c) low intensity found in HaCaT and in MEF. As it was mentioned, high intensity was observed exclusively in tumor cells, thus suggesting sCrot-Cy3 molecule accumulation; however, no morphological signs of cell death were observed after 24 h (Supplemental Videos S1–S8). 

The interest in 3D cell culture in cancer research is growing over time and considered closer to real life models, especially in cancer stem cells research [45,46,47]. sCrot also penetrates effectively into melanospheres (3D model), as well as into the cells growing in suspension. The sCrot in 3D cancer cell models may be useful for studying interactions between cancer and stromal cells providing a model for tumor microenvironment investigation and for testing new therapeutics.

According to current knowledge [6], besides sCrot, only buforin IIb presents cancer cells-specific penetrating ability, however it is toxic and affects the viability of normal cells [20]. Our group previously showed that at nCrot is non-toxic for normal cells as well as non-embryotoxic at a micromolar range [1]. However, we assessed the viability of the tumor and non-tumor cells used in present study after incubation with sCrot at different doses. The uptake assay demonstrates that sCrot do not display any cytotoxic activity for 24 h, which was also confirmed by the measurement of the activity of dehydrogenase in cells, which is directly proportional to the number of living cells (Figure 6B). Tumor cells specific non-toxic CPPs are of great importance for effective cancer treatment because of requirement of anti-cancer drugs target delivery that help to minimize side effects on normal cells [48,49]. 

Our group demonstrated nCrot selective penetration into the tumors in two different in vivo models [19,50]. However, preferential accumulation of nCrot in tumor cells versus non-tumor cells as well as preferential co-localization of sCrot with internal membranes have never been evidenced. This data strongly suggests that sCrot that is non-toxic for both tumor and non-tumor cells and when used as a carrier of anti-cancer drugs, it may be able deliver more copies of the drug of interest inside the tumor cells. Additionally, it can be used potentially as a fluorescent probe for accurate surgical removal of tumor cells aiming to ovoid possible reoccurrence of tumor growth. In basic research, sCrot labeled cells isolated from the tumor may be further processed by sorting to separate specific tumor cell populations using additional appropriate markers, for instance for cancer stem and progenitor cells and terminally differentiated cancer cells. After the sorting, these cells might be used for single-cell transcriptomic and proteomic analysis. 

## 4. Materials and Methods

### 4.1. Crotamine 

Synthetic crotamine (sCrot) was purchased from the Smartox Company (Saint-Egrève, France) and to introduce the fluorescent label, the protein was N­terminally conjugated with Cy3­reactive dye (sCrot­Cy3) by the manufacturer.Native crotamine was acquired as described in the Supplemental Material (Section 1).

### 4.2. Cell Lines and Culture Condition

SK­MEL­28—human melanoma, B16-F10—murine melanoma and SKBR3—human mammary tumor cell lines were cultured in Dulbecco’s modified Eagle medium (DMEM High Glucose (LGC Biotecnologia, Cotia, Brazil) supplemented with 10% System Approved Fetal Bovine Serum (LGC Biotecnologia) and 2 mM l-glutamine (LGC Biotecnologia) and 1% penicillin­streptomycin (LGC Biotecnologia); HaCaT cells were cultured under the same conditions. Human melanoma cells—A2058 were cultured in RPMI 1640 Medium (LGC Biotecnologia) supplemented with 10% System Approved Fetal Bovine Serum and 2 mM l-glutamine and 1% penicillin­streptomycin. Cells were routinely passaged and cultured at 37 °C in a humidified incubator with 5% CO_2_.

Isolation of primary mouse embryo fibroblasts (MEFs) was performed by harvesting embryos from female mice 13–14 days pregnant and assayed enzymatic digestion by using 0.25% trypsin-EDTA. The cells were then cultured in DMEM Ham’s F12 medium (LGC Biotecnologia) supplemented with 10% System Approved Fetal Bovine Serum and 2 mM l-glutamine and 1% penicillin-streptomycin. All experimentation with animals was performed in accordance to the guidelines of the Institutional Ethics Committee (COBEA—Colégio Brasileiro de Experimentação Animal) under protocol number 903/12.

Jurkat-E6 lymphocyte-derived leukemia cell was cultured in RPMI supplemented with 10% Bovine Fetal Serum, 2 mM l-glutamine and 1% penicillin/streptomycin. The cells were kept at 37 °C in a humidified incubator with 5% CO_2_.

Human peripheral blood mononuclear cells (PBMC) were isolated by Ficoll-Hypaque density gradient centrifugation separation method (density 1.077 g/mL—Sigma Aldrich, St. Louis, EUA). To do so, approximately 20 mL of blood was collected from healthy volunteers (Plataforma Brasil/CEP 1.806.596) in tubes with sodium heparin. The blood was diluted in physiological saline solution (0.9%) at a ratio of 1:1 with blood. Then this blood was conditioned in a conical tube containing Ficoll-Paque, in a 1:3 proportion to blood. The mononuclear cells were cultured at 37 °C in a humidified incubator with 5% CO_2_ in RPMI.

### 4.3. Generation of Melanoma Spheroids and sCrot-Cy3 Uptake 

Melanoma spheroids were generated via the ‘hanging drop’ method [51] incorporating 250 melanoma cells per 25 μL of RPMI medium placed on the lid of a non-adhesive petri dish containing PBS. Spheroids were incubated at 37 °C, 5% CO_2_. Every third day, 8 μL of the medium per drop was exchanged. After 15 days, spheroids were harvested and maintained in a non-adhesive petri dish. Next, sCrot (1 μM) was added onto the spheroids and after 5 min of incubation the images were acquired using an Eclipse Ti–S instrument (Nikon, Tokyo, Japan).

### 4.4. Uptake Experiments

#### 4.4.1. Confocal Laser Scanning Microscopy

First, the cells (3 × 10^4^/well–24 well plate) were incubated with 0.5 μM of a cell internal membrane marker DiOC_6_(3) (3,3′dihexyloxacarbocyanine iodide) for few seconds and then washed with PBS several times. Next, the living cells were incubated with 1 μM of sCrotCy3 at different times points (5 min, 6 h and 24 h), they were then washed with PBS and fixed with 3.4% of paraformaldehyde for 20 min followed by several washes with PBS. Zeiss LSM 510 Meta laser scanning confocal microscope equipped with inverted Zeiss Axiovert 200 M stand (Carl Zeiss GmbH, Jena, Germany) was used for visualization at 60× magnification. Images of DiOC_6_(3) were acquired in the FITC channel using 490/20 nm excitation, 525/36 nm emission. Images of nuclei in the DAPI channel were acquired using 350/50 nm excitation and 455/58 nm emission. Images of sCrot-Cy3 were acquired using 550/70 nm excitation and 570/30 nm emission. All images were acquired using LSM 510 software (Carl Zeiss GmbH, Jena, Germany). For measuring the co-localization area between internal cell membranes and sCrot, BioImage XD (Dresden, Germany) software was used including the statistical methods of Pearson [37], Manders [38], Costes [39] and Li [40]. All the background fluorescence values were subtracted from experimental data. This experiment was executed three times and the average of the results was used for statistical analyses.

#### 4.4.2. High-Content Imaging: Time Lapse 

The cells were seeded on a 24 well plate at a concentration of 1 × 105 cells per well. On the next day, sCrot-Cy3 (1 µM) was added onto the cells. The suspension cells (PBMC and Jurkat-E6) were sedimented by a quick spin. Live cell high-content imaging was performed using IN CELL Analyser 2200 microscope system (GE Healthcare, Little Chalfont, UK) placed in a temperature-controlled room (37 °C). Ten tiles were imaged per well at 40× magnification. Time-lapse images were obtained every 10 min for 24 h. For measuring, the fluorescence intensities of the uptake of sCrot-Cy3 in the time-lapse images, 144 images were analyzed for each cell line using ImageJ software (National Institutes of Health, Bethesda, MD, USA) was used. This experiment was executed twice, and the mean of the results was used for statistical analyses.

### 4.5. Cell Viability Assay

The cells were seeded at a density of 2.5 × 10^4^ into a 96-well plate and treated with sCrot (not Cy3 labeled) at different concentration (0–25 µM) for 24 h. Cell viability was assessed using the Cell Counting Kit-8 (CCK-8, Dojindo Molecular Technologies, Tokyo, Japan) which is based on the dehydrogenase activity detection in viable cells. The CCK-8 solution was added in a ratio 1:20 to every well for the last 2 h, and the optical density (OD) value of each well at 450 nm wavelength was recorded. The assay was conducted three times with three replicates each time. 

### 4.6. Statistical Analyses 

Statistical significance (*p* values) was assessed using the two-tailed Student’s test (*t*-test). Error bars, where shown, designate standard deviations.

## 5. Conclusions

In conclusion, the present data certify the use of the sCrot as a cancer targeting CPP equal to nCrot, as well as demonstrates its potential biotechnological application as a tool for tumor cell investigation in basic and applied research.

## Supplementary Materials

The following are available online. Figure S1: nCrot-Cy3 uptake and co-localization with internal cellular membranes stained by DiOC_6_(3) observed in tumor and non-tumor cells; Figure S2: Western blot of native and synthetic crotamine performed with anti-crotamine antibody; Figure S3: Representative MALDI-TOF mass spectra of nCrot-Cy3, nCrot, sCrot-Cy3 and sCrot-Cy3. Table S1: Natural toxins derived CPPs, their potential intracellular targets and their mechanism of action in vitro and in vivo; Table S2: Brief comparison of coefficients used to estimate co-localization with their meaning, ranges of values, and use. Videos: Video S1 A2058, Video S2 SK-MEL-28, Video S3 B16 F10, Video S4 SKBR3, Video S5 HaCaT, Video S6 MEF, Video S7 PBMC and Video S8 Jurkat.

## References

[1] Kerkis A., Kerkis I., Radis-Baptista G., Oliveira E.B., Vianna-Morgante A.M., Pereira L.V., Yamane T. (2004). Crotamine is a novel cell-penetrating protein from the venom of rattlesnake *Crotalus durissus terrificus*. FASEB J..

[2] Kerkis A., Hayashi M.A.F., Yamane T., Kerkis I. (2006). Properties of cell penetrating peptides (CPPs). IUBMB Life.

[3] Kerkis I., Hayashi M.A.F., Prieto da Silva A.R.B., Pereira A., De Sa Junior P.L., Zaharenko A.J., Radis-Baptista G., Kerkis A., Yamane T. (2014). State of the art in the studies on crotamine, a cell penetrating peptide from South American rattlesnake. Biomed. Res. Int..

[4] Nascimento F.D., Hayashi M.A.F., Kerkis A., Oliveira V., Oliveira E.B., Radis-Baptista G., Nader H.B., Yamane T., Tersariol I.L.D., Kerkis I. (2007). Crotamine mediates gene delivery into cells through the binding to heparan sulfate proteoglycans. J. Biol. Chem..

[5] El-Sayed A., Futaki S., Harashima H. (2009). Delivery of macromolecules using arginine-rich cell-penetrating peptides: Ways to overcome endosomal entrapment. AAPS J..

[6] Raucher D., Ryu J.S. (2015). Cell-penetrating peptides: Strategies for anticancer treatment. Trends Mol. Med..

[7] Bechara C., Sagan S. (2013). Cell-penetrating peptides: 20 years later, where do we stand?. FEBS Lett..

[8] Gupta B., Levchenko T.S., Torchilin V.P. (2005). Intracellular delivery of large molecules and small particles by cell-penetrating proteins and peptides. Adv. Drug Deliv. Rev..

[9] Li H., Tsui T.Y., Ma W.X. (2015). Intracellular delivery of molecular cargo using cell-penetrating peptides and the combination strategies. Int. J. Mol. Sci..

[10] Munyendo W.L., Lv H., Benza-Ingoula H., Baraza L.D., Zhou J. (2012). Cell penetrating peptides in the delivery of biopharmaceuticals. Biomolecules.

[11] Rathnayake P.V., Gunathunge B.G., Wimalasiri P.N., Karunaratne D.N., Ranatunga R.J. (2017). Trends in the binding of cell penetrating peptides to siRNA: A molecular docking study. J. Biophys..

[12] Su Y.C., Doherty T., Waring A.J., Puchala P., Hong M. (2009). Roles of arginine and lysine residues in the translocation of a cell-penetrating peptide from c-13, p-31, and f-19 solid-state NMR. Biochemistry.

[13] Zorko M., Langel U. (2005). Cell-penetrating peptides: Mechanism and kinetics of cargo delivery. Adv. Drug Deliv. Rev..

[14] Fosgerau K., Hoffmann T. (2015). Peptide therapeutics: Current status and future directions. Drug Discov. Today.

[15] Esteve E., Mabrouk K., Dupuis A., Smida-Rezgui S., Altafaj X., Grunwald D., Platel J.C., Andreotti N., Marty I., Sabatier J.M. (2005). Transduction of the scorpion toxin maurocalcine into cells—Evidence that the toxin crosses the plasma membrane. J. Biol. Chem..

[16] Boisseau S., Mabrouk K., Ram N., Garmy N., Collin V., Tadmouri A., Mikati M., Sabatier J.M., Ronjat M., Fantini J. (2006). Cell penetration properties of maurocalcine, a natural venom peptide active on the intracellular ryanodine receptor. Biochim. Biophys. Acta-Biomembr..

[17] Shahbazzadeh D., Srairi-Abid N., Feng W., Ram N., Borchani L., Ronjat M., Akbari A., Pessah I.N., De Waard M., El Ayeb M. (2007). Hemicalcin, a new toxin from the iranian scorpion *Hemiscorpius lepturus* which is active on ryanodine-sensitive Ca^2+^ channels. Biochem. J..

[18] Gurrola G.B., Capes E.M., Zamudio F.Z., Possani L.D., Valdivia H.H. (2010). Imperatoxin a, a cell-penetrating peptide from scorpion venom, as a probe of Ca^2+^-release channels/ryanodine receptors. Pharmaceuticals (Basel).

[19] Nascimento F.D., Sancey L., Pereira A., Rome C., Oliveira V., Oliveira E.B., Nader H.B., Yamane T., Kerkis I., Tersariol I.L.S. (2012). The natural cell-penetrating peptide crotamine targets tumor tissue in vivo and triggers a lethal calcium-dependent pathway in cultured cells. Mol. Pharm..

[20] Lim K.J., Sung B.H., Shin J.R., Lee Y.W., Kim D.J., Yang K.S., Kim S.C. (2013). A cancer specific cell-penetrating peptide, BR2, for the efficient delivery of an scFv into cancer cells. PLoS ONE.

[21] Ponnappan N., Chugh A. (2017). Cell-penetrating and cargo-delivery ability of a spider toxin-derived peptide in mammalian cells. Eur. J. Pharm. Biopharm..

[22] Kerkis I., de Brandão Prieto da Silva A.R., Pompeia C., Tytgat J., de Sá Junior P.L. (2017). Toxin bioportides: Exploring toxin biological activity and multifunctionality. Cell Mol. Life Sci..

[23] Valdivia H.H., Kirby M.S., Lederer W.J., Coronado R. (1992). Scorpion toxins targeted against the sarcoplasmic-reticulum Ca^2+^-release channel of skeletal and cardiac-muscle. Proc. Natl. Acad. Sci. USA.

[24] Hayashi M.A.F., Nascimento F.D., Kerkis A., Oliveira V., Oliveira E.B., Pereira A., Radis-Baptista G., Nader H.B., Yamane T., Kerkis I. (2008). Cytotoxic effects of crotamine are mediated through lysosomal membrane permeabilization. Toxicon.

[25] Schwartz E.F., Capes E.M., Diego-Garcia E., Zamudio F.Z., Fuentes O., Possani L.D., Valdivia H.H. (2009). Characterization of hadrucalcin, a peptide from *Hadrurus gertschi* scorpion venom with pharmacological activity on ryanodine receptors. Br. J. Pharmacol..

[26] Radis-Baptista G., de la Torre B.G., Andreu D. (2008). A novel cell-penetrating peptide sequence derived by structural minimization of a snake toxin exhibits preferential nucleolar localization. J. Med. Chem..

[27] Radis-Baptista G., de la Torre B.G., Andreu D. (2012). Insights into the uptake mechanism of NrTP, a cell-penetrating peptide preferentially targeting the nucleolus of tumour cells. Chem. Biol. Drug Des..

[28] Jha D., Mishra R., Gottschalk S., Wiesmuller K.H., Ugurbil K., Maier M.E., Engelmann J. (2011). Cylop-1: A novel cysteine-rich cell-penetrating peptide for cytosolic delivery of cargoes. Bioconjug. Chem..

[29] Ponnappan N., Budagavi D.P., Chugh A. (2017). Cylop-1: Membrane-active peptide with cell-penetrating and antimicrobial properties. Biochim. Biophys. Acta-Biomembr..

[30] Ohkura M., Furukawa K., Tu A.T., Ohizumi Y. (1994). Calsequestrin is a major binding protein of myotoxin alpha and an endogenous Ca^2+^ releaser in sarcoplasmic reticulum. Eur. J. Pharmacol..

[31] Tu A.T., Morita M. (1983). Attachment of rattlesnake venom myotoxin a to sarcoplasmic reticulum: Peroxidase conjugated method. Br. J. Exp. Pathol..

[32] Guidotti G., Brambilla L., Rossi D. (2017). Cell-penetrating peptides: From basic research to clinics. Trends Pharmacol. Sci..

[33] Vanregenmortel M.H.V. (1993). Synthetic peptides versus natural antigens in immunoassays. Ann. Biol. Clin..

[34] Kolomin T., Shadrina M., Slominsky P., Limborska S., Myasoedov N. (2013). A new generation of drugs: Synthetic peptides based on natural regulatory peptides. Neurosci. Med..

[35] Edmondson R., Broglie J.J., Adcock A.F., Yang L.J. (2014). Three-dimensional cell culture systems and their applications in drug discovery and cell-based biosensors. Assay Drug Dev. Technol..

[36] Sztiller-Sikorska M., Koprowska K., Jakubowska J., Zalesna I., Stasiak M., Duechler M., Czyz M.E. (2012). Sphere formation and self-renewal capacity of melanoma cells is affected by the microenvironment. Melanoma Res..

[37] Pearson K. (1906). On certain points connected with scale order in the case of the correlation of two characters which for some arrangement give a linear regression line. Biometrika.

[38] Manders E.M.M., Verbeek F.J., Aten J.A. (1993). Measurement of colocalization of objects in dual-color confocal images. J. Microsc.-Oxf..

[39] Costes S.V., Daelemans D., Cho E.H., Dobbin Z., Pavlakis G., Lockett S. (2004). Automatic and quantitative measurement of protein-protein colocalization in live cells. Biophys. J..

[40] Li Q., Lau A., Morris T.J., Guo L., Fordyce C.B., Stanley E.F. (2004). A syntaxin 1, Galpha(o), and N-type calcium channel complex at a presynaptic nerve terminal: Analysis by quantitative immunocolocalization. J. Neurosci..

[41] Manders E.M.M., Stap J., Brakenhoff G.J., Vandriel R., Aten J.A. (1992). Dynamics of 3-dimensional replication patterns during the s-phase, analyzed by double labeling of DNA and confocal microscopy. J. Cell Sci..

[42] Hoppe A., Christensen K., Swanson J.A. (2002). Fluorescence resonance energy transfer-based stoichiometry in living cells. Biophys. J..

[43] Bolte S., Cordelieres F.P. (2006). A guided tour into subcellular colocalization analysis in light microscopy. J. Microsc.-Oxf..

[44] Schenberg S. (1959). Geographical pattern of crotamine distribution in the same rattlesnake subspecies. Science.

[45] Wang C.Y., Tang Z.Y., Zhao Y., Yao R., Li L.S., Sun W. (2014). Three-dimensional in vitro cancer models: A short review. Biofabrication.

[46] Bielecka Z.F., Maliszewska-Olejniczak K., Safir I.J., Szczylik C., Czarnecka A.M. (2016). Three-dimensional cell culture model utilization in cancer stem cell research. Biol. Rev. Camb. Philos. Soc..

[47] Penfornis P., Vallabhaneni K.C., Janorkar A.V., Pochampally R.R. (2017). Three dimensional tumor models for cancer studies. Front. Biosci..

[48] Geisler I., Chmielewski J. (2009). Cationic amphiphilic polyproline helices: Side-chain variations and cell-specific internalization. Chem. Biol. Drug Des..

[49] Martín I., Teixidó M., Giralt E. (2010). Building cell selectivity into CPP-mediated strategies. Pharmaceuticals (Basel).

[50] Pereira A., Kerkis A., Hayashi M.A.F., Pereira A.S.P., Silva F.S., Oliveira E.B., Prieto da Silva A.R.B., Yamane T., Radis-Baptista G., Kerkis I. (2011). Crotamine toxicity and efficacy in mouse models of melanoma. Expert Opin. Investig. Drugs.

[51] Foty R. (2011). A simple hanging drop cell culture protocol for generation of 3D spheroids. J. Vis. Exp..

