# Supplementary material for: Co-Localization of Crotamine with Internal Membranes and Accentuated Accumulation in Tumor Cells
Source: Molecules. 2018 Apr 20;23(4):968. doi: 10.3390/molecules23040968 (PMC6017820; doi:10.3390/molecules23040968)
Supplement: Supplementary file 1 [file molecules-23-00968-s001.zip › molecules-279284-SI.pdf]

molecules

Article

Co-Localization of Crotamine With Internal Membranes and Accentuated Accumulation in Tumor Cells.

Nicole Caroline Mambelli-Lisboa[1,2#], Juliana Mozer Sciani[2,3], Alvaro Rossan Brandão Prieto da Silva[1,2], Irina Kerkis[1,2#]

List of the material:

1. Experimental procedure
2. Figure S1. nCrot-Cy3 uptake and co-localization with internal cellular membranes stained by $DiOC_6(3)$ observed in tumor and non-tumor cells.
3. Figure S2. Western blot performed with anti-crotamine antibody (rabbit) 1:4000.
4. Figure S3. Representative MALDI-TOF mass spectra of crotamines.
5. Table S1. Natural toxins derived CPPs, their potential intracellular targets and their mechanism of action in vitro and in vivo.
6. Table S2. Brief comparison of coefficients used to estimate co-localization with their meaning, ranges of values, and use.

Supplemental Data

Experimental procedure

1. Native Crotamine

Native and pure crotamine was kindly provided by Dr. Eduardo Brandt, Faculty of Medicine, University of São Paulo (Ribeirão Preto, Brazil). The purification from *Crotalus durissus terrificus* venom was performed as previously described[4]. Briefly, the venom was diluted in ammonium formate buffer and the major venom component (crotoxin) was eliminated by slow speed centrifugation. Dropwise addition of Tris base in the solution was used to raise the pH of the supernatant to 8.8 and it was then applied to CM-Sepharose FF column (1.5 × 4.5 cm; Amersham Biosciences, Buckinghamshire, UK). Subsequently, the column was washed and crotamine was recovered, dialyzed, lyophilized and stored (nCrot).

2. Biological crotamine activity in vivo

C57BL/6 mice were obtained (n=6) from the animal breeding facility of the Butantan Institute. Food and water were available to the animals throughout the experiment. All experimentation with animals was performed in accordance to the guidelines of the Institutional Ethics Committee (COBEA – Colégio Brasileiro de Experimentação Animal) under protocol number 903/12.

The biological activity of synthetic crotamine was compared to the native protein isolated from the venom by injecting intraperitoneally (IP) into mice in sublethal doses corresponding to 2.5 mg of toxin/kg body mass, which provokes the hind limb paralysis (GONÇALVES et al, 1956). The mice were divided in two groups, three animals were injected with sCrot and three with nCrot.

3. Labeling of native crotamine with Fluorescent Dye

Fluorescent crotamine (nCrotCy3) was prepared by using the Cy3reactive dye (GEHealthcare, UK) following the instructions of the manufacturer. Briefly, 1 mg of native crotamine was dissolved in 0.5 mL of 0.1 M sodium bicarbonate buffer and added to 1 mg of the reactive dye. The reaction was incubated overnight at 4°C for 1 hour with continuous stirring. After labeling, the fluorescent dye remaining (not conjugated) was eliminated by a reversed phase chromatography, using a HPLC system (Shimadzu Co. Japan), in a C18 column (Ascentis, 15 cm x 4.6 mm). The elution was performed at 5–40% gradient of solvent B (90% acetonitrile/H_2O with 0.1% formic acid) over solvent A (H_2O with 0.1% formic acid) in 40 min, at a constant flow rate of 0.5 mL/min. A PDA detector monitored the eluted content, and according to the chromatographic profile, fraction was manually collected, lyophilized and stored at -20°C the fractions were analyzed by MALDITOF (AXIMA performance, Shimadzu), at positive and reflectron mode, with alpha-cyano-4-hydroxycinnamic acid as matrix. Data were obtained at a range of 500 – 10000 m/z.

4. Synthetic and native crotamine detection by Western blot

A sample of synthetic crotamine (10 µg/lane) was submitted to 15% SDSPAGE under reducing conditions[13]. The native crotamine (10 µg/lane) was used as a positive control. The gel was placed in the electro blot apparatus adjacent to nitrocellulose paper in buffer, as described by Towbin and collaborators[14] and transferred for 90 min at 0.85 mA/cm2. The membrane was blocked with 5% milk in pH 7.4 phosphate buffered saline and incubated with rabbit polyclonal antibody against whole crotamine (diluted 1:4000) (Oguiura et al., 2000). The immunoreactive proteins were detected using peroxidase labeled AntiRabbit antibody (diluted 1:4000).

5. Uptake experiments

First, the cells were incubated with 0.5 µM of a cell internal membrane marker $DiOC_6(3)$ (3,3′dihexyloxacarbocyanine iodide) for few seconds and then washed with PBS several times. Next,

the living cells were incubated with 1 μM of nCrotCy3 at different times points (5 min, 6 hours and 24 hours), they were then washed with PBS and fixed with 3.4% of paraformaldehyde for 20 minutes followed by several washes with PBS. Zeiss LSM 510 Meta laser scanning confocal microscope equipped with inverted Zeiss Axiovert 200M stand (Carl Zeiss GmbH, Jena, Germany) was used for visualization. Images of $DiOC_6(3)$ were acquired in the FITC channel using 490/20 nm excitation, 525/36 nm emission. Images of nuclei in the DAPI channel were acquired using 350/50 nm excitation and 455/58 nm emission. Images of nCrot-Cy3 were acquired using 550/70 nm excitation and 570/30 nm emission. All images were acquired using LSM 510 software (Carl Zeiss GmbH, Jena, Germany). This experiment was executed three times and the average of the results was used for statistical analyses.

Supplemental Figures

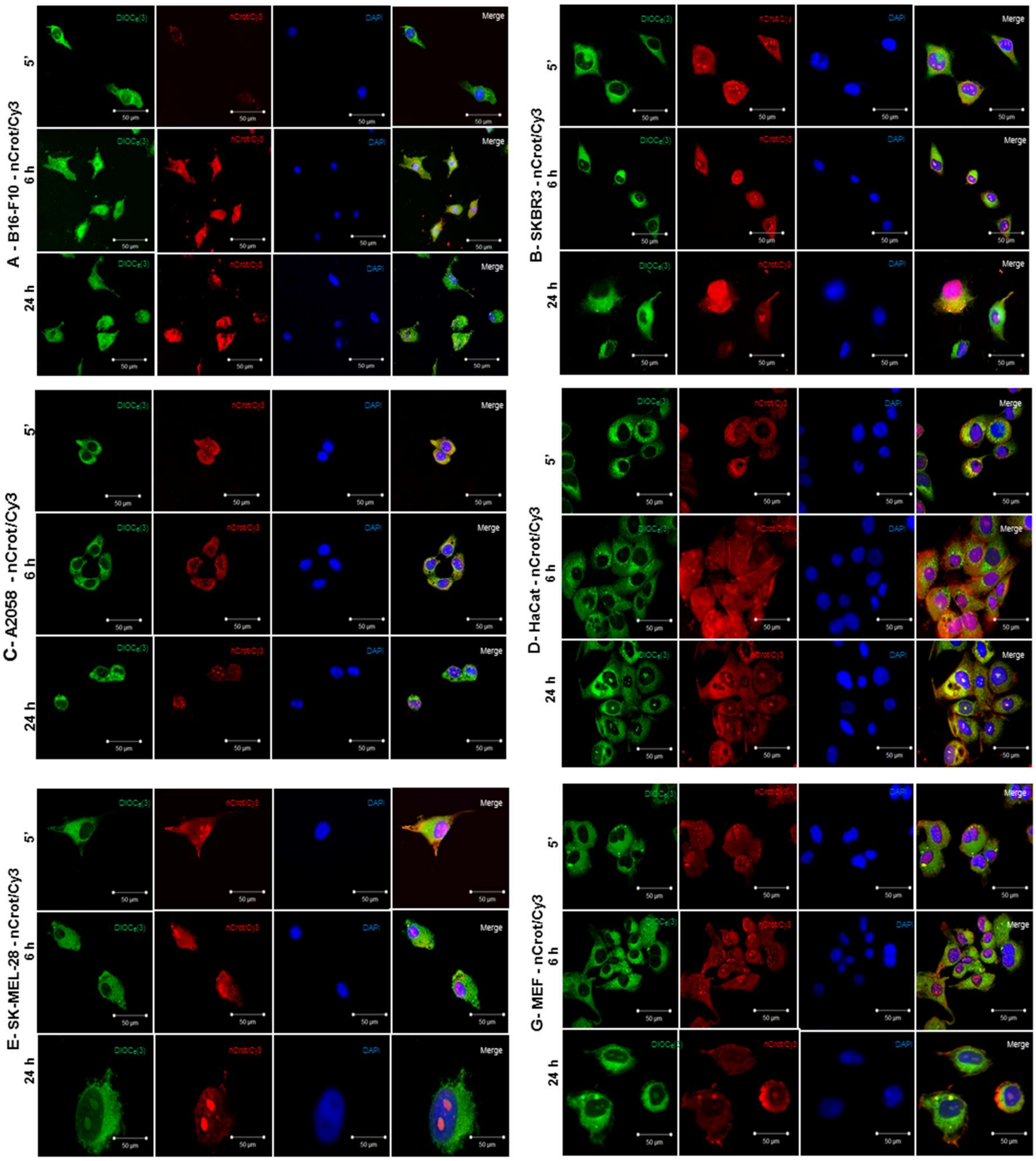

Figure S1. nCrot-Cy3 uptake and co-localization with internal cellular membranes stained by $DiOC_6(3)$ observed in tumor and non-tumor cells. A- nCrot-Cy3 uptake after 5 min, 6 and 24 hours in murine B16-F10 cells (B) in human SKBR3 cells; (C) in human A2058 cells; (D) in human HaCaT cell; in human SK-MEL-28 cells; (G) in murine MEF cells. Internal cellular membranes stained by $DiOC_6(3)$ (green); Nuclei stained by DAPI (blue) Scale bars (A-F3) = 50µm. (A-F3) = Fluorescent confocal microscopy (FCM).

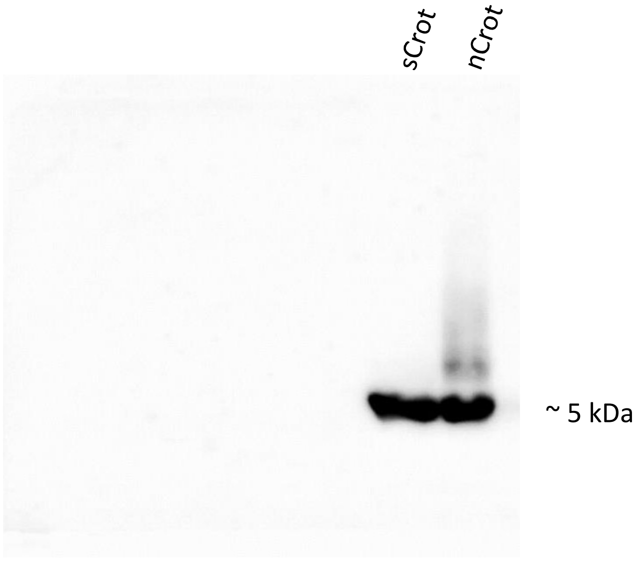

Figure S2. Western blot of native and synthetic crotamine performed with anti-crotamine antibody (rabbit) 1:4000. The immunoreactive proteins were detected using peroxidase labeled Anti¬Rabbit (1:10000)

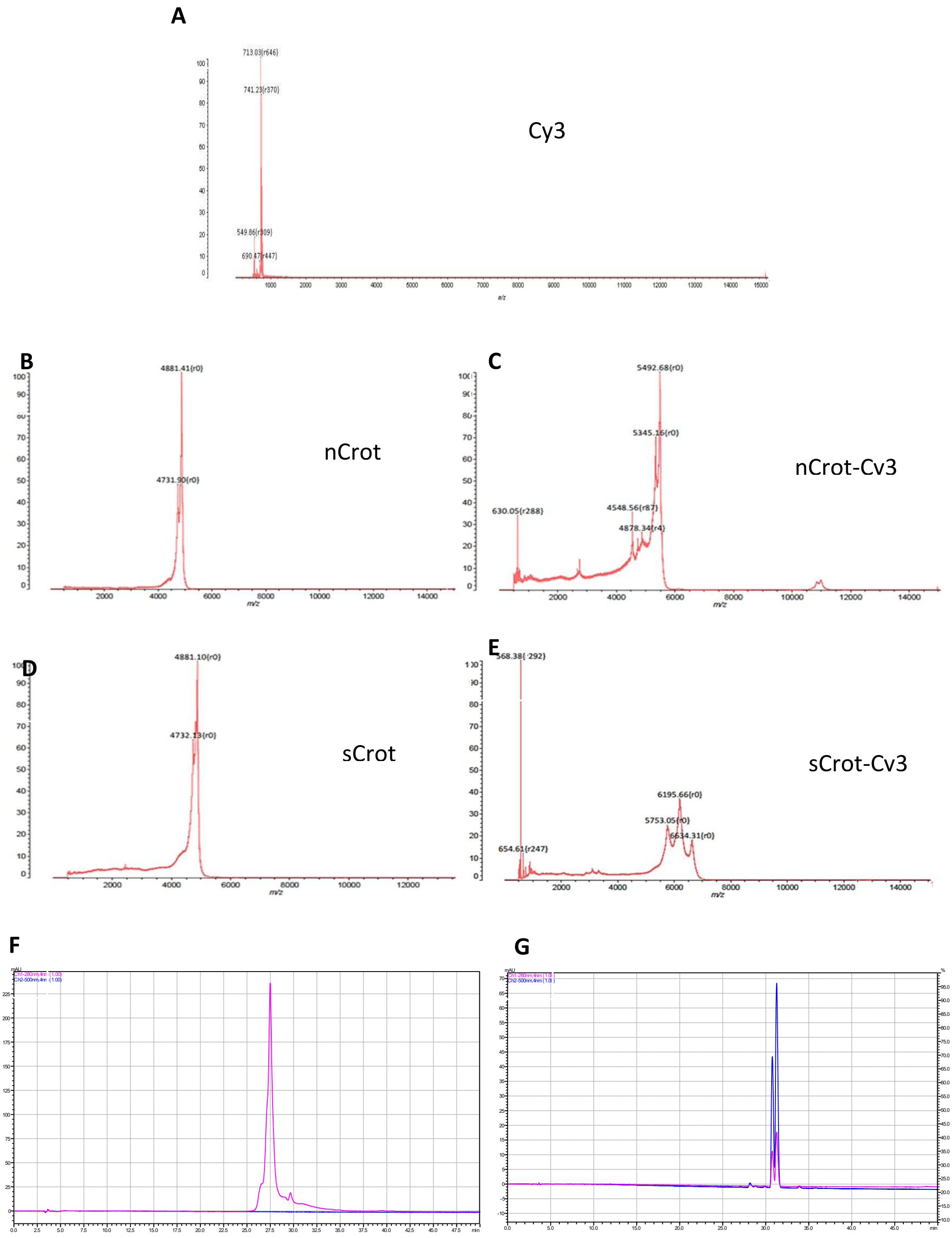

Figure S3. Representative MALDI-TOF mass spectra shown in the range of m/z 500 to 1500 of (A) Cy3 dye control fraction (B) purified native crotamine, (C) purified native crotamine/Cy3 labeled, (D) synthetic crotamine and (E) synthetic crotamine /Cy3 labeled. Figures F and G show the purification of Cy3-crotamine by RP-HPLC analyzed in both wavelengths, 280 nm (pink) and 500 nm (blue). (F) crotamine, is observed only by 280 nm and not by 500 nm (the Cy3 wavelength) and (G) the labeled crotamine, with a retention time shift, indicating the increase of the hydrophobicity after the labeling, as expected. It is notable the presence of two peaks – one correspondent to Cy3-crotamine and other the dye, both observed by 280 and 500 nm

Supplemental Tables

Table S1. Natural toxins derived CPPs, their potential intracellular targets and their mechanism of action *in vitro* and *in vivo*.

Peptide	Origin	Target	Mechanism of action	*In vitro* results	*In vivo* results
**Crotamine (1)**	Rattlesnake (*Crotalus durissus terrificus)*	Nucleus and cytoplasm	n/a	Marker of actively proliferating cells. Noncitotoxic and did not affect the pluripotency of ES cells	Did not affect the development of mouse embryos
**Maurocalcine (MCa) (15,16)**	Scorpion (*Scorpio maurus maurus)*	Ryanodine receptor	Binds directly to the skeletal muscle ryanodine receptor, and induces long-lasting Ca^{2+} channel opening	Show no toxicity on HEK293 cell	n/a
**Hemicalcin (17)**	Scorpion (*Hemiscorpius lepturus*)	Ryanodine receptor	Activation of ryanodine receptors	Induce the release of Ca^{2+} from internal stores	n/a
**Imperatoxin A (IpTxa) (18,23)**	Scorpion (*Pandinus imperator)*	Ryanodine receptor	Activation of Ca^{2+} channels	Release of Ca^{2+} from the sarcoplasmatic reticulum in ventricular myocyte mouse.	Crossing cell membrane to alter the Ca^{2+} releasing *ex* vivo
**Crotamine (19)**	Rattlesnake (*Crotalus durissus terrificus*)	Lysosome, endoplasmic reticulum and mitochondria	Ca^{2+} release and loss of mitochondrial membrane potential	Increase of free intracellular Ca^{2+} concentration	Selective uptake into tumor nodule
**Latarcin–derived peptide (LDP) (21)**	Spider (*Lachesana tarabaevi)*	Nucleus and cytoplasm	Penetrates into cells through endocytosis and destabilizes the endosomal membrane.	Translocates across cell membrane in HeLa cells, with no cytotoxicity and delivery macromolecular protein inside the cells.	n/a

**Crotamine (24)**	Rattlesnake (*Crotalus durissus terrificus*)	Lysosome	Release of cathepsin and an increase of caspase activity	Accumulation in lysosomes, affecting the proliferative activity and leading to cell death	Uptake into distinct mice tissues, sucha as liver, skeletal muscle, brain and kidney
**Hadrucalcin (25)**	Scorpion (*Hadrurus gertschi*)	Ryanodine receptor	Activation of ryanodine receptors	Induce the release of Ca^{2+} from internal stores	Binding of hadrucalcin with ryanodine receptors in dog and rabbit
**NrTP (Nucleolar-Targeting Peptide) (26,27)**	Crotamine-derived peptides	Nucleolus	Clathrin-dependent route as the primary NrTP1 uptake mechanism	Selective nucleolar uptake at HeLa, BxPC-3 and BT-474 cells. In murine neuroblastoma (N2A) cells, was a partial uptake on the membrane and endocytic structures	n/a
**CyLOp-1 (28,29)**	Crotamine-derived peptides	Cytoplasm	The presence of cysteine residues in the peptide play a major role in conferring cell-penetrating	Antimicrobial activity, induction of caspase-3 activity and selectively delivery	n/a
**Myotoxin alpha (30)**	Rattlesnake (*Crotalus viridis viridis*)	Calsequestrin / Heavy fraction of sarcoplasmic	Activation of calcium release in skeletal muscle	Release of Ca^{2+} from storage compartments	Muscular necrosis
**Myotoxin alpha (31)**	Rattlesnake (*Crotalus viridis viridis*)	Binding in membranes of sarcoplasmic reticulum and possible colocalization with Ca^{2+}-ATPase	Partial antibody blocking against Ca^{2+}-ATPase	Release of Ca^{2+} from storage compartments	Muscular necrosis

Table S2. Brief comparison of coefficients used to estimate co-localization with their meaning, ranges of values, and use.

Method	Value range	Colocalisation if …
Pearson's coef r	+1= coloc<br>0= random<br>-1= exclusion	tends to 1
Manders' coef M1 (or M2)	0= 0% of Ch1 colocalize with Ch2<br>1= 100% of Ch1 colocalize with Ch2	tends to 1
Costes (P-value)	P<95% = no coloc<br>P≥95% = coloc	≥95%
Li (ICQ)	0.5= coloc<br>0= random<br>- 0.5= exclusion	Tends to 0.5
